# Special Issue “Adenosine Receptors as Attractive Targets in Human Diseases”

**DOI:** 10.3390/ph14020140

**Published:** 2021-02-10

**Authors:** Daniela Catarzi, Flavia Varano, Vittoria Colotta

**Affiliations:** Dipartimento di Neuroscienze, Psicologia, Area del Farmaco e Salute del Bambino, Sezione di Farmaceutica e Nutraceutica, Universita’ degli Studi di Firenze, Via Ugo Schiff 6, 50019 Sesto Fiorentino (Firenze), Italy; flavia.varano@unifi.it (F.V.); vittoria.colotta@unifi.it (V.C.)

The idea of promoting this Special Issue arises from the desire to witness the multidisciplinary efforts that are currently in progress to provide new insights into the pathophysiological role of adenosine. The different articles and reviews herein reported highlight the continuing interest in this research field that still offers new challenges every day. 

About a hundred years have passed since the role of adenosine as an extracellular signaling molecule was proposed [1]. From then on, many researchers have worked extremely hard to study and collect thousands of pieces of experimental evidence on its biological relevance. Adenosine exerts its physiological roles by interacting with four G protein-coupled adenosine receptors (ARs), termed A_1_, A_2A_, A_2B_, and A_3_ [2]. All ARs affect cAMP levels by the modulation of adenylyl cyclase. However, other effector systems have been proposed [3,4]. ARs are widely distributed throughout the body, and their activation modulates a large variety of effects in different tissues and organs. In fact, the concentration of adenosine increases under unfavorable conditions to counteract tissue damage. However, despite its homeostatic regulator role [3], there are conditions in which the chronic and excessive production of adenosine can be harmful [5]. In fact, elevated adenosine levels and/or the upregulation of ARs have been detected in many pathological conditions [6]. Thus, the development of biologically active compounds that interfere with adenosine signaling at different levels has been and still is of interest. 

In the past, the detection of ARs has been facilitated and made possible by the development of specific radioligands for the different ARs. In fact, since the discovery of the biological relevance of adenosine, the development of specific AR probes, such as pharmacological tools for receptor characterization, has been constantly evolving. Starting from radioligands [2], nowadays increased importance is given to fluorescent and covalent ligands for many purpose [2,7]. In this Special Issue of *Pharmaceuticals*, the article by Federico and co-workers reviewed specific chemical probes for ARs, which are useful for the application to structural biology studies, drug discovery, and diagnostics [8]. Currently, application of the methodology for covalent probes represents an important tool for stabilizing target proteins to obtain X-ray crystal structure [2,7].

Availability of potent and selective radioligands for ARs has been fundamental for supporting the drug discovery process. In fact, the first screening of newly synthesized compounds is, in general, consistent in rapid radioligand binding assays. Many different classes of ligands were evaluated at ARs, and to date several recurring structural features have been identified for defining the affinity or efficacy of compounds for a specific AR [6]. Among the large variety of scaffolds which have been investigated for the development of new AR antagonists, the thiazolo[5,4-d]pyrimidine core has been recently proposed by Varano and co-workers [9,10]. Their most recent contribution is included in the current Special Issue, where a SAR study on a series of new thiazolo[5,4-d]pyrimidines as A_2A_AR inverse agonists is reported [11]. It is worth noting that this series of ligands also comprises some previously published derivatives showing affinity values for the A_2A_ subtype in the femto-molar range and with an inverse agonists profile [10]. 

While antagonists are characterized by a large structural variability, the agonists profile has been long associated with an adenosine-like structure [6]. In contrast, more recent studies have reported some non-nucleoside compounds belonging to the 6-aminopyridine-3,5-dicarbonitrile series that showed different degrees of efficacies at the different ARs. Some of these ligands, thought to be lacking the ribose portion, have significant affinity and efficacy for human ARs. The review of Dal Ben and co-workers reported in this Special Issue [12] describes developments in this field from 2000 to today. Starting from the first non-selective agonists, many amino-3,5-dicyanopyridine derivatives endowed with high potency and improved selectivity for specific ARs have been developed. Besides summarizing the influence of the structural features on biological activity, the work also reviewed the synthetic pathways yielding to the target compounds and the molecular modeling studies at the suitable AR binding pocket. Some structurally related compounds are described in the article by Catarzi and co-workers that is reported in this Special Issue. New amino-3,5-dicyanopyridines spanning from pan ligands to combined A_1_/A_2B_ partial agonists potentially useful for treating diabetes and related complications were reported [13].

In recent times, much progress has been made toward the elucidation of the roles of ARs in physiological as well as pathological conditions. In fact, it has emerged that adenosine modulates a wide variety of pathophysiological process at the level of both the cardiovascular and the central nervous systems. Moreover, it is involved in immune system dysregulations, and also in tumor onset and progression [6]. Thus, each AR has been taken into consideration as a potential target for the development of ligand-based therapies against a wide range of pathologies. In particular, a number of AR agonists have emerged as useful agents to counteract cardiovascular disorders and related diseases, such as hypercholesterolemia and diabetes [14,15]. The article by Wolska and collaborators reported in this Special Issues [16] describes the synergistic effects of nine AR agonists (previously known to inhibit platelet aggregation [17]: PSB0777, CGS21680, MRE0094, 2-chloroadenosine, CV1808, HE-NECA, NECA, regadenoson, and UK423,097) with purinergic P2Y12 receptor antagonists in inhibiting platelet function. This approach could be an innovative strategy to counteract blood platelet hyperactivity that yields arterial thrombosis with consequent acute and often lethal cardiovascular insult. 

While the implication of adenosine in cardiovascular disease is well documented [14], its role in the immune system is still controversial. In fact, experimental evidence supports the use of antagonists as well as agonists of all ARs in the control of immune diseases [18]. In particular, the prolonged immune response together with the dysregulation of the immunomodulatory adenosine signaling can contribute to the development of autoimmune disorders. In the review of Magni and co-workers reported in this Special Issue, the authors provide a general overview of the role of adenosine in the modulation of the immune system, with special attention to the potential adenosine-based therapies for autoimmune disorders [19]. Particular focus is on the A_3_AR subtype, which may represent a very promising therapeutic target due to its overexpression in inflammatory cells [20].

To summarize, the investigation of ARs and their ligands has grown rapidly over the years and has had a great impact on the drug discovery process. In particular, the dichotomy of the adenosine system and the consequent controversies still existing with regard to the pathophysiological roles of ARs have made the goal more challenging and stimulating. Nowadays, the potential for compounds to interfere with adenosine signaling is very encouraging, since some of them, having emerged as potential innovative therapeutics, are likely to move from their role of candidates to that of drugs for clinical use [21]. 

Let us speculate that the ambitious goal of advancing adenosine receptor ligands into drugs to improve human health is getting closer and more real. This is what every researcher working in this field would like to see realized.

Finally, we would like to thank all the authors that have contributed to this Special Issue on adenosine and its receptors, as well as the reviewers that have accepted the commitment to revise the quality of manuscripts, thus improving their scientific relevance.

## Data Availability

Data sharing not applicable.
